# The Impact of Physical Exercises with Elements of Dance Movement Therapy on Anthropometric Parameters and Physical Fitness among Functionally Limited Older Nursing Home Residents

**DOI:** 10.3390/ijerph20053827

**Published:** 2023-02-21

**Authors:** Natalia Wołoszyn, Justyna Brożonowicz, Joanna Grzegorczyk, Justyna Leszczak, Andrzej Kwolek, Agnieszka Wiśniowska-Szurlej

**Affiliations:** 1 Institute of Health Sciences, College of Medical Sciences, University of Rzeszów, 35-310 Rzeszów, Poland; 2 DONUM CORDE Rehabilitation and Medical Care Center, 36-060 Budy Głogowskie, Poland

**Keywords:** elderly, physical health, anthropometry, nursing homes

## Abstract

Changes in the composition of the body mass of functionally limited older patients may contribute to a decrease in functional fitness and the development of chronic diseases. This research aimed to assess the differences in anthropometric parameters and physical fitness of older patients, over the age of 65, in a 12-week clinical intervention study. Method: The study participants were nursing home inhabitants aged 65–85 who were functionally limited. Persons meeting the inclusion criteria were assigned to one of the three groups: Group 1–basic exercises/BE group (n = 56); Group 2—physical exercises with elements of dancing/PED group (n = 57); Group 3—control group/CO group (n = 56) routine care. The data were collected at the beginning of the study and at the 12-week mark. The outcome was observed for hand grip strength (HGS), arm curl test (ACT), Barthel Index (BI), Berg Balance Scale (BBS), triceps skin fold (TSF), waist-to-hip-ratio (WHR), and arm muscle area (AMA). Results: The study included 98 women and 71 men. The average age of the participants was 74.40 years. The analysis of the effects of the 12-week exercise program showed the greatest changes in HGS, ACT, and BI in the exercise groups, especially in the PED group compared to the BE group. Statistically significant differences in the examined parameters of the PED vs. BE vs. CO groups were demonstrated in favour of the exercising groups. In conclusion, a 12-week program of group physical exercises, both PED and BE, improves physical fitness indicators and anthropometric indicators.

## 1. Introduction

Changes in the composition of the body mass of older patients may contribute to a decrease in functional fitness and the development of chronic diseases. Scientific research confirms a strong relationship between a decrease in muscle strength, physical fitness, and the loss of lean body mass [1]. A decrease in skeletal muscle mass and function is seen in older patients and is associated with sarcopenia, which is a health concern [2]. One of the main risk factors for sarcopenia is a low level of physical activity along with a decline in muscle fibers that begins in middle age. The decrease in muscle mass and strength increases the risk of fractures, the quality of life decreases, and independent living becomes more difficult [2,3] With age, the distribution and function of adipose tissue reorganize, reaching a peak by the age of 70 and then beginning to decline. In the following decades of life, excess adipose tissue is deposited in the epicardium, bone marrow, liver, and muscles, which leads to the loss of lean body mass and organ dysfunction, and consequently to obesity and a decrease in the fitness of older patients [4,5].

During the aging process, there are changes in the composition of body mass, which cause a decrease in bone mineral density and muscle mass and an increase in fat mass [6,7]. In an aging society, one of the most significant metabolic diseases is osteoporosis, which leads to the weakening of bone microstructures, reducing bone mineral density (BMD), and increasing the risk of fractures [8,9]. As indicated by Tsutsumi et al., it is important to systematically assess anthropometric parameters in older patients, as their results are easy to obtain and correlate with the risk of mortality and the duration of hospitalization [10]. In addition, Colleluori and Villareal emphasized that it is important to conduct research that takes the impact of physical exercise on anthropometric indicators among functionally limited older patients into account [11]. Combining various interventions/exercises in old age gives the opportunity to improve functional fitness. The use of aerobic exercises can achieve improvements in peak oxygen consumption as well as have beneficial effects on blood pressure, lipids, glucose tolerance, bone density, depression, and quality of life [12]. A properly designed resistance training program for older patients should include an individualized periodic approach involving the performance of 2–3 sets of 1–2 multi-joint exercises per major muscle group, reaching an intensity of 70–85% of 1-repetition maximum (1RM) 2–3 times per week, and include strength training performed at higher speeds in moderate-intensity concentric movements (i.e., 40–60% 1RM). Resistance training programs for older patients should follow the principles of individualization, periodization, and progression. A properly designed resistance training program can counteract age-related changes such as contractile function, atrophy, and morphology of aging human skeletal muscles. In addition, it can increase strength, muscle power, and neuromuscular functioning in older patients [13]. Exercise of the hypertrophy type is the main modality that aims to increase skeletal muscle size [14]. However, in some cases, even 3-month resistance training was not effective in achieving a practically significant nor statistically significant increase in lean mass compared to sham-intervention in healthy older participants [15]. According to the World Health Organization (WHO), older adults should engage in at least 150–300 min of moderate-intensity aerobic physical activity (PA) per week, or at least 75–150 min of vigorous-intensity aerobic physical activity, or an equivalent combination of moderate-intensity and vigorous-intensity aerobic physical activity per week. Two days a week, they should do moderate or higher-intensity muscle-strengthening exercises involving all of the major muscle groups, as they provide additional health benefits.

To increase functional capacity and prevent falls, as part of their weekly physical activity, older adults should engage in multi-component physical activity 3 or more days a week that emphasizes functional balance and moderate-to-high-intensity strength training [16]. Low functional fitness is a common and complex geriatric condition characterized by the failure of multiple systems in the body along with reduced capacity for recovery. The functionally limited older patients are particularly exposed to the risk of adverse health effects, deterioration of cognitive functioning, physical disability, and, as a consequence, hospitalization and institutionalization [17]. Despite the fact that the decline in psychophysical health is inextricably linked to the aging process, regular physical activity contributes to slowing down this process and improving functional fitness in everyday life [18]. In the literature on the subject, there is a great deal of scientific evidence confirming the beneficial effects of physical activity (PA) on the body in old age. Pepera et al. studied forty older patients, long-term care residents, who were assigned to two groups: intervention (IG) and control (CO). The IG group performed a twice-weekly, two-month, multi-component exercise program consisting of exercises for functional mobility, balance, muscle strength, and flexibility; the CO did not perform any movement intervention. During the two-month exercise program, systolic blood pressure and function improved in the older patients and residents of long-term care facilities in the group IG [19]. Similarly, Thomas et al., reviewing randomized studies among people over 65 years of age, note a similar observation. They report that balance can be improved by various means of exercise training and that promoting physical activity in older people is essential [20].

Despite the widely publicized benefits of PA, a significant proportion of older patients do not meet the minimum and recommended PA levels. Therefore, effective interventions are needed to increase physical activity in older patients. One such form is the introduction of dance elements into standard exercise programs. According to scientific reports, this action combines the benefits of physical activity with improving mood, thereby affecting the quality of life [21]. Elements of choreotherapy, such as music and movement exercises or movement improvisations, effectively improve balance and functional fitness and are conducive to social integration. Group exercises with elements of dance alleviate the feeling of social isolation and give a sense of belonging to a group [22]. In addition, dance, regardless of style, can significantly improve muscle strength, endurance, balance, and other aspects of functional fitness in older patients [23]. Any style of dance can induce positive functional adaptations in older people, especially in terms of balance. A result of dancing can also be the improvement of metabolism. Dance could be a potential movement intervention to promote health benefits for aging individuals [24].

Existing research does not provide sufficient exercise guidelines for nursing home residents to improve existing health resources and prevent or delay the loss of physical function in functionally limited older patients [25].

In their systematic review of the scientific literature, Fernández-Argüelles et al. noted the positive impact of factors such as balance, gait and dynamic mobility, strength, and physical capacity on the risk of falls; however, based on the evidence, the authors were unable to confirm that dance has significant benefits on these factors due to some unsatisfactory aspects of the research, such as methodological quality, low sample size, lack of homogeneity with respect to variables and measurement tools, and the existence of variability in the study design and type of dance [26]. Similarly, Rodrigues-Krause et al. claim that there are no randomized clinical trials (RCTs) evaluating metabolic and anthropometric outcomes related to dance interventions [24].

Despite the knowledge about the impact of physical activity on aging, there are few reports in the available literature on the influence of physical exercises with elements of dancing on physical fitness and anthropometric indicators. To our knowledge, this is the first study to focus on older adults and tailor interventions for people with functional limitations. In addition, in the assessment, we combine physical indicators and anthropometric parameters. Therefore, this research project aimed to assess the differences in anthropometric measurements and physical fitness of a sample of Polish older patients over the age of 65 in a 12-week clinical intervention study.

## 2. Materials and Methods

### 2.1. Study Design and Sample Description

The study was designed in accordance with consort guidelines for reporting randomized controlled trials. The study was conducted in 5 randomly selected nursing homes in Podkarpacie (south-eastern Poland). The sample size was estimated from a priori power analysis to detect statistically significant effects of exercise [27]. The sample size was chosen according to the Cohen method, using standard assumptions: 0.05 for significance level, 0.8 for the power of the test, and 0.5 for effect size which accounts, according to Cohen, for medium effect size. Sample size calculation for the main outcome measure was based on changes in HGS scores.

The inclusion criteria for participation in the study are as follows: age between 65 and 85 years, cognitive functioning status enabling participants to execute instructions and answer questions, moderate limitations in functional fitness (Barthel score in the range of 21–75 points), no serious diseases that would make it impossible to participate in the study, giving informed consent to participate in the study, and living in a nursing home. The exclusion criteria included the inability to perform active movements with the upper and lower limbs and the presence of unstable internal diseases (recent myocardial infarction, uncontrolled arrhythmias, acute congestive heart failure, unstable angina, acute pulmonary embolism or pulmonary infarction, uncontrolled hypertension, uncontrolled diabetes mellitus, acute systemic infections accompanied by fever, body aches, or swollen lymph nodes) [28,29,30]. 

**Figure 1:**
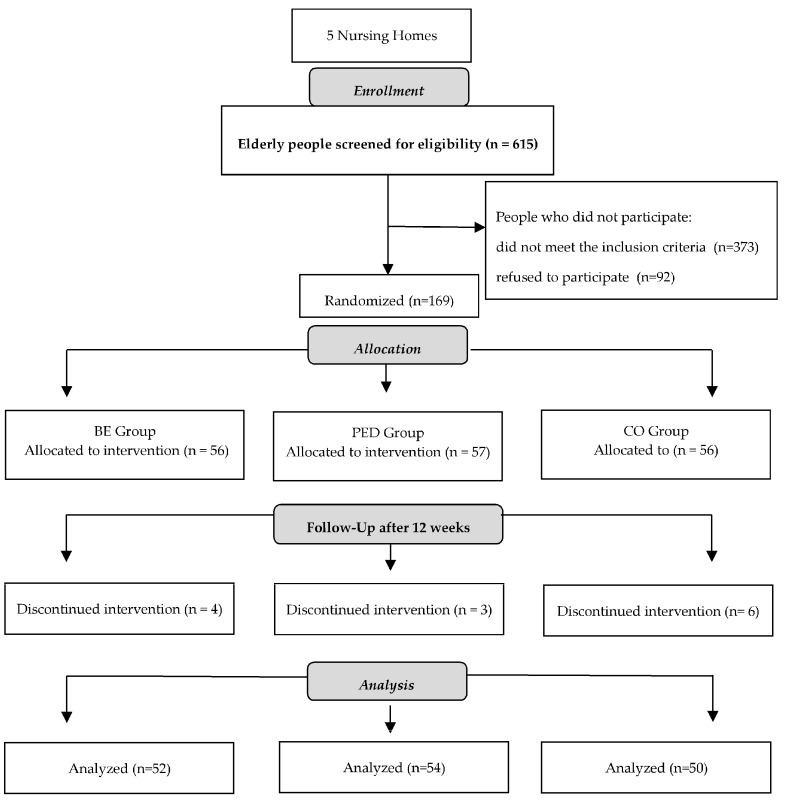
Flow diagram of participants throughout the study.

People who met the criteria for participation in the study were randomly assigned to one of three groups: basic exercises (BE)—56 people, physical exercises with elements of dancing (PED)—57 people, and control group (CO)—56 people. Randomization was carried out by implementing the stratified method with the use of the statistical package R 3.2.2. Three-in-one blocks were randomized, which made it possible to obtain an even distribution of older patients in the studied groups. The order of randomization was determined using a computerized schedule of random numbers. An independent biostatistician implemented randomization, hid the block size from the executive module, and used randomly mixed block sizes. The person conducting the randomization was not involved in the recruitment process or assessment and did not carry out interventions in any of the groups. Of the 169 randomized participants, 156 (92.3%) completed the study after 12 weeks. The completion rate of the study was as follows: BE group, 52 people; PED group, 54 people; CO group, 50 people. The most common reasons for withdrawal were related to loss of interest in the study and change of place of residence. No adverse events related to participation in the exercise programs were reported. The stages of including participants in the study and assigning them to particular groups are presented in the CONSORT flow diagram (Figure 1).

The study was approved by the Bioethics Committee of the University of Rzeszów (No. 9/11/2017). In accordance with the Declaration of Helsinki, all participants were informed about the purpose and procedure of the study and gave their informed consent to participate in the study. In the absence of informed consent, the participant was excluded from the further research procedure.

The study protocol has been registered with the Sri Lanka Clinical Trials Registry (SLCTR/2018/014).

### 2.2. Intervention

In each of the groups, a different program of action was implemented.

Group 1 Basic exercises (BE)—exercises were performed in a sitting position, in groups of 6–8 people. The exercises were carried out without background music and without the use of accessories. In accordance with the recommendations of the World Health Organization (WHO) regarding physical activity in older patients, the program included exercises based on aerobic exercise, strengthening exercises for the upper and lower limbs, trunk exercises, as well as balance exercises [16]. Each session consisted of a warm-up (10 min), a main part (20 min), and a final part (5 min). Each exercise was preceded by instructions. As part of the warm-up, simple upper and lower limb exercises were performed, leading to faster breathing and heart rate and preparing muscles and joints for the main exercises (e.g., marching in place, circulation in the shoulder joints, turning the arm, flexion and extension in the elbow joints, and opening and clenching the fist). Aerobic warm-up exercises were supplemented with breathing exercises. In the main part, exercises were performed to strengthen the upper and lower limbs (e.g., lifting the torso with the hands on the seat, slowly moving to a standing position, or tilting the torso forward and backward in a sitting position) and for balance and coordination (e.g., alternating weight on the right and left buttock and alternate lifting of the opposite arm and leg). A total of 6–12 repetitions of each exercise were performed in two series (the first week started with 6 repetitions, and in each subsequent week of implementation, the program was performed with one more repetition of each exercise). The pace of exercise was adjusted to the abilities and well-being of those exercising and did not exceed 11–13 points on the Borg scale of exercise intensity. Between the series of each exercise, there was a 30 s break in which breathing exercises were performed. In the final part of each session, calming and relaxing exercises were performed (e.g., calm breaths combined with exercises maintaining the range of motion in the joints—slow bends and twists of the trunk) the purpose of which was to normalize the heart rhythm [31,32].

Group 2: Physical exercises with elements of dancing (PED)—in this group, exercises were carried out in a sitting position. In the initial part of the classes, a warm-up (5 min) was carried out based on simple movements of the upper and lower limbs and simple movement improvisations to the rhythm of music. The exercises began with rhythmic hand clapping and walking in place to the rhythm of the music. Then, those exercising imitated the physiotherapist conducting the classes and performed movements that simulated, for example, driving a car, flying an airplane, rustling trees, and picking fruit. The main part (20 min) was based on simple movements and dances performed in a sitting position. The movements to the music were mainly based on hand clapping, waving the hands, and stamping the feet. During the sessions, dance movements to the rhythm of music were implemented, e.g., cha-cha, Zumba, jive, macarena, and Hawaiian dance. Each musical arrangement consisted of 6–8 movement sequences appropriate to the type of music. Before starting the exercises, the physiotherapist taught the participants the appropriate sequence of movements without the use of music. During the exercises, equipment was also used: canes, TheraBands, and balls. Each program was structured in such a way as to include elements of strengthening (e.g., stretching a TheraBand or squeezing a ball) and balancing exercises (e.g., tilting the trunk forward and diagonally with raised arms). In order to keep the exercisers interested, the music and exercise routines were changed weekly for the first 6 weeks, with the sequence repeated in succession for the next 6 weeks. The nature of the music and its tempo were selected depending on the current perceptual and motor abilities of older patients exercising. After each session, the subjective intensity of exercise in the study group was assessed using the Borg scale. The intensity of the exercises was planned so as not to exceed 11–13 points on the Borg scale. At the end of each session, there was a short calming part (5 min) in which breathing exercises and simple stretching exercises were performed to calm relaxing music [33].

In both groups 1 and 2, the program was carried out twice a week for 30 min in the morning over a 12-week period. Before the start of each exercise session, the participants were asked about their current well-being, and each participant had their blood pressure and pulse measured in order to qualify for exercise on a given day. The classes were conducted by a physiotherapist trained in the research procedure and experienced in working with older patients.

Group 3: Control group (CO)—in this group, no therapeutic program was implemented. People assigned to this group followed their standard daily schedule.

### 2.3. Outcome Measures

All data were collected before the intervention and after the 12-week exercise program.

#### 2.3.1. Socio-Demographic Data, Data on Health, and Nutrition

In order to characterize the study group, socio-demographic data such as age, sex, marital status, education level, and time of stay in a nursing home were collected. Data on coexisting diseases in the examined participants were obtained on the basis of the analysis of the medical records of the nursing home residents. The height and weight of the participants were also measured, and then the BMI (kg/m^2^) was determined. The BMI value was classified in accordance with the standards proposed by the Committee of Diet and Health, according to which the normal body weight for people over 65 corresponds to a BMI in the range of 24–29 kg/m^2^, underweight—below 24 kg/m^2^, and overweight—above 29 kg/m^2^ [34]. On the basis of interviews with the participants, data on the subjective and objective assessment of their nutritional status (the number of meals, the amount of fluids, and the intake of particular types of products) were also collected. The survey form contained questions about chronic (long-term) diseases, e.g., cardiovascular diseases, including chronic stroke, myocardial infarction, controlled hypertension, and atherosclerosis under control.

#### 2.3.2. Muscle Strength (Hand Grip Strength HGS)

The measurement was carried out using a hand dynamometer (JAMAR PLUS + Digital Hand Dynamometer, Patterson Medical). The measurement was performed in a sitting position on a chair without armrests, with the elbow joint of the dominant arm bent at 90 degrees, the forearm in a neutral position, and the wrist extended between 0 and 30 degrees. The participant was asked to clench their hand as much as possible and hold it for 6 s. The procedure was repeated three times with a one-minute rest between trials. The result was given in kilograms and as the average of the three measurements [35].

#### 2.3.3. Manual Endurance of the Upper Limb (Arm Curl Test ACT)

The measurement was carried out in a sitting position on a chair without armrests, with feet flat on the floor. The measurement was performed with a weight of 2 kg for women and 3.5 kg for men. The participant’s task was to flex the forearm with the dominant hand in supination and return to the starting position (extension of the forearm in pronation). The result of the test was the number of repetitions in 30 s [36].

#### 2.3.4. Functional Assessment (Index Barthel IB)

The scale assesses the participant’s degree of independence in basic daily activities. It includes 10 activities such as dressing, bathing, mobility, eating, using the toilet, moving around, and controlling urine and stool excretion. Depending on the degree of independence in each of the assessed activities, the participant was assessed at the level of 0–15 points, with the higher score indicating greater independence. A total of 100 points can be obtained on the scale. On the basis of the total score obtained on the scale, the participant can be classified into one of three categories of need for care [37].

#### 2.3.5. Body Balance Assessment (Berg Balance Scale BBS)

The scale assesses the participant’s static and dynamic balance in 14 trials. In each of the trials, the participant’s balance is assessed on a scale of 0–4, with a higher assigned point value indicating a better level of balance during the performance of a given trial. The results obtained in each trial are added together, and in total the participant may obtain 56 points [38].

#### 2.3.6. Triceps Skinfold (TSF)

The measurement was carried out using calipers. In order to carry out the measurement, a point halfway between the posterior edge of the acromion process and the ulnar process of the left hand was determined. Then, at this point, the skin-fat fold was pulled vertically with the thumb and forefinger and its measurement was made by applying the arms of the calipers and tightening them until they were fully stabilized. The result was read to the nearest 1 mm [39].

#### 2.3.7. The Waist-to-Hip Ratio (WHR)

The value was determined on the basis of the ratio of the waist circumference measured in centimeters, measured at the height of the last rib, to the maximum circumference of the hips. The normal values were 0.71–0.85 for women and 0.78–0.94 for men. Higher values were interpreted as abdominal obesity and lower values as gluteofemoral obesity [40].

#### 2.3.8. Arm Muscle Area (AMA)

In order to determine the AMA value, the mid-arm circumference was measured. The distance between the ulnar process and the shoulder projection was determined. The circumference of the arm was measured at a point halfway along that distance. Next, the arm muscle circumference was determined according to the formula: arm muscle circumference = arm circumference-ח (triceps skin fold thickness). The AMA value was determined according to the formula: arm muscle area (AMA) = arm muscle circumference^2^ /ח [41,42].

### 2.4. Statistical Analysis

Statistical analysis of the collected material was performed using the Statistica 13.3 package. The database and the graphical elaboration of the results were prepared in Microsoft Excel and Microsoft Word. Descriptive characteristics were presented as means and standard deviations, or numbers and percentages.

The model of elaboration of results for dependent trials (to examine the assessment of effects before and after therapy) and for independent trials (between groups) was adopted. To examine the effects before and after therapy (measuring scale of the dependent variable of a quantitative nature), the t-Student test for dependent samples was used, and in case of lack of normal distribution of differences in dependent variables, the Wilcoxon non-parametric test was used. Differences between the groups for differences in the studied parameters were assessed using t-Student tests for independent samples (independent variable on two levels) or one-way ANOVA (independent variable on at least three levels). When the assumption of normality of distribution (verified by the Shapiro–Wilk test) of the dependent variables was not met, the following non-parametric tests were used: Mann–Whitney U or Kruskal–Wallis. Statistically significant results for differences in at least three groups were supplemented with post-hoc Tukey tests or post-hoc multiple comparisons of mean ranks for all samples. Pearson’s chi-square tests of independence were used in the analysis if the data represented a non-ranked category (measuring scale of nominal variables).

Table 1 description: Sociodemographic and clinical data of the participants were taken into account and differences between the three groups (BE; PED; CO) were examined. Quantitative data meeting the criteria of normality of distribution within groups (tested by Shapiro–Wilk test) and homogeneity of variance (tested by Levene’s test) were qualified to use the one-way ANOVA variance analysis test. If one of the above assumptions was violated, the Kruskal–Wallis test was used. Significantly statistical results for the one-way analysis of variance were completed using the Tukey post-hoc test, and the Kruskal–Wallis post-hoc test was used for the Kruskal–Wallis test. The significance of differences between the two nominal variables was calculated using Pearson’s Chi square test.

Table 2 description: A statistical model was adopted in which the effect of intervention between Measure I and Measure II was tested. For the normality of the distributions of differences (tested by the Shapiro–Wilk test), the Student’s *t*-test for dependent samples was used. If the assumption of the normality of the distribution of differences was violated, the non-parametric Wilcoxon test was used.

Table 3 and Table 4 descriptions: Firstly, the difference between the 1st and 2nd survey was calculated (2nd measurement minus 1st measurement). It was then examined whether these differences from the two measurements differed significantly between the groups. It was decided that the study groups be treated individually by pairing them (PED vs. BE; PED vs. CO; BE vs. CO); therefore, the Student’s *t*-test for independent samples was used if the assumption of normality of distribution (checked with the Shapiro–Wilk test) and homogeneity of variance (checked with the Levene’s test) was met. If the normality of the distribution was not observed in the study groups, the decision was made to use the non-parametric Mann–Whitney test.

## 3. Results

The study included 98 women (57.99%) and 71 (42.01%) men. The average age of the participants was 74.40 years (SD = 7.45). For the entire study group the average number of daily consumed meals was 3.11 per person (SD = 0.79). According to the subjective assessment of nutritional status, 77 people (45.56%) believed that they were malnourished, and 54 participants (31.95%) did not report any disorders in their eating. In the study group, 141 people (83.40%) were right-handed, and 28 participants (16.60%) indicated the left hand as dominant. The mean grip strength for the right hand was 12.81 kg (SD = 5.27) and 11.20 kg for the left hand (SD = 4.41). The average BBS score for all groups was 12.41 points (SD = 5.61), and for the BI scale 54.03 points (SD = 11.69).

Before starting the exercise programs, all study groups were comparable with each other in terms of socio-demographic characteristics (except education level), functional fitness, including upper limb strength, endurance and balance, muscle mass index, and skin fold index. The participants from the CO group showed a lower level of education than the BE and PED groups (CO < BE, PED). Before the intervention, the BMI and body mass scores in the CO group were higher than in the BE group (CO < BE; CO < BE, PED, respectively). In terms of arm muscle area (AMA), the results were statistically significantly higher in the PED group than in the BE and CO groups (PED < BE, CO). However, before the start of the study, WHR results were significantly higher in the PED group compared to the BE group. The demographic data of the participants and the basic parameters are summarized in Table 1.

After 12 weeks of intervention, the measured parameters were compared with the baseline data. The analysis of the results showed that in the BE and PED group there was a moderately significant increase in BBS, ACT, HGS, AMA parameters and a decrease in TSF and BMI values. However, in the CO group there was a decrease in BBS, ACT, HGS and an increase in TSF and BMI. Detailed data are presented in Table 2.

### Mean Difference Scores for Each Group across Time

The analysis of the effects of the 12-week exercise program showed the greatest changes in HGS, ACT, BI and BMI in the exercise groups, especially in the PED group compared to the BE group. However, in the CO group, a decrease in the value of most of the parameters studied was observed. In the BE group, after 12 weeks of physical exercise, an improvement was demonstrated: in HGS R by 5.54 kg and in HGS L by 6.60 kg; in the ACT test by 2.56 repetitions; and in the BBS scale by an increase of 1.90 points. After the intervention, a decrease in body weight by an average of 2.50 kg was shown, which in BMI showed a decrease of 0.96 points, and an average decrease in WHR of −0.03. The greatest positive changes after 12 weeks of intervention were shown in the PED group: HGS R by an average of 6.54 kg and HGS L by 7.74. For the PED group, the number of repetitions in the ACT test increased by 3.63, and in the BBS scale the number of points increased by an average of 2.67. There was a decrease in body weight by an average of 3.50 kg and BMI by 1.51 points. However, an increase in the WHR index by 0.02 points and AMA area by 885.32 was found. In the CO group, after 12 weeks of observation, there was a decrease in HGS R and L, ACT, BI, BBS, and AMA, while an increase in anthropometric indicators such as body mass, BMI, triceps skin fold, and WHR was noted. The mean difference scores for each group after 12 weeks are shown in Table 3.

After 12 weeks of exercise and observation, statistically significant differences were found between the PED and BE groups in all parameters examined. After the intervention period, statistically significant differences were found between functional status and anthropometric indicators between the PED and CO groups. No statistically significant difference was found between the PED group and the CO group in WHR results. Statistically significant differences in the examined parameters evaluating functionally limited older patients in the BE vs. CO groups were demonstrated. The differences between the groups after 12 weeks from the start of the study are presented in Table 4.

## 4. Discussion

Lack of physical stimulation causes health disorders and functional impairment, which negatively affects functioning in everyday life and dependence on third parties. Declining health and physical limitations may make it difficult for some older patients to participate in exercise programs designed for people without mobility limitations [43]. In this study, changes in anthropometric parameters and physical fitness in a group of functionally limited older patients were assessed after 12 weeks of participation in a PA program or with no intervention. The obtained results indicate that the program, both in the form of general fitness exercises (basic exercises) and in the form of physical exercises with elements of dancing, improves the functional fitness and anthropometric parameters of older patients.

Our research used group exercises designed for functionally limited older patients living in nursing homes and demonstrated improvements in physical fitness. The results of a systematic review by Shakeel et al. support the effectiveness of group exercise. The authors propose the implementation of this type of program as an effective and economical way to provide nursing home residents with physical and social health benefits [44]. The results of a meta-analysis by Jansen et al. confirm the potential of group intervention programs to increase physical activity among residents of nursing homes. [45]

In our study, we have shown that the addition of elements of dancing has a positive effect on the physical and functional fitness of functionally limited older patients. In addition, music during physical exercise can reduce the perceived effort and improve mood, as well as reduce anxiety and the symptoms of depression. Rucello et al. point out that music is an important tool to support the involvement of older people in physical exercise [46].

The results of our studies showed an improvement in muscle strength and manual endurance of the upper limb in the exercise groups and a decrease in HGS R and L and ACT in the CO group. Similar results were obtained by Chiu et al. In a randomized control study, the authors used their own program of exercises in a sitting position among 64 residents of long-term care homes. The program lasted 12 weeks and the exercises were carried out twice a week. Music was used to increase the motivation of the participants to exercise. The researchers also showed a statistically significant improvement in hand grip strength in the intervention group compared to the control group [47]. Chen et al. investigated the effectiveness of a group exercise program based on the use of elastic bands in resistance training. Elderly people in wheelchairs performed exercises three times a week for 40 min. The results showed high effectiveness of the intervention in terms of improving functional fitness, including improvement of hand grip strength, upper and lower body flexibility, and lung function [48]. Falconer et al. and Williams et al. showed that manual dexterity is strongly correlated with the degree of independence in older people. Limitation of manual dexterity also causes a limitation in the scope of instrumental activities of daily living and an increase in dependence on other people [49,50]. Similar results were obtained by Scherder et al., who showed that a reduction of manual dexterity is associated with increases in dependency in everyday activities, institutionalization, and mortality [51].

The analysis of the impact of a 3-month exercise program on everyday activities assessed using the Barthel scale showed improvement in the exercising groups (BE and PED group). There were no significant changes in the CO group after 12 weeks of observation. Similar results were obtained by Venturelli et al., whose study aimed to assess the impact of upper body training on the fitness of older women with mobility limitations. The results of the authors’ research showed a significant improvement in activities of daily living and no changes in the control group [52]. In a meta-analysis, Crocker et al. showed that physical rehabilitation has a positive effect on improving the performance of everyday activities of older patients living in nursing homes. The authors believe that exercise is an important factor in reducing disability among older people in long-term care [53]. The study by Machacova et al. assessed the impact of a 3-month intervention with elements of dance among older patients in a nursing home. The authors claim that relatively simple dance-based exercises can reduce physical decline [54]. Murrock et al. based their research on the theory of music, mood, and movement. The intervention was aimed at evaluating the impact of dance exercises on the functional fitness of older patients. The authors demonstrated improved functional fitness following a 12-week exercise program [55].

On the basis of our own research, there was an improvement in balance in the BE and PED groups. In the CO group, deterioration was observed in the area under study. On the basis of a meta-analysis, Chou et al. showed the positive effect of various types of physical interventions on improving balance among frail older patients. Despite the existence of multi-directional exercise programs, the most effective one for the elderly population has not been clearly identified [56]. Telenius et al. and Grönstedt et al. evaluated the impact of exercise programs on static and dynamic balance in elderly people living in nursing homes. They showed a statistically significant improvement in this parameter between the exercise group and the control group [57,58]. Eyigor et al. assessed the effect of folk dance on balance in a randomized study. After an 8-week dance program, the examined group of older women had improved their physical fitness and balance [59].

We showed that after 12 weeks of exercise, a decrease in body weight was observed in the study group, and, consequently, a decrease in BMI; greater changes were noted in the PED group (by −1.51 on average) than in the BE group (by −0.96 on average). Similar results were obtained by Valdés-Badilla et al. after 16 weeks of physical exercise among the entire study group [60]. Research published by Merchant et al. on BMI and waist circumference showed that a high BMI was associated with better functional and cognitive status [61]. On the other hand, LaCroix et al. showed that loss of physical fitness strongly correlates with very high BMI [62]. An earlier study by Colleluori et al. confirms that physical exercise is beneficial in reducing body weight while maintaining muscle mass. In addition, the authors suggest training protocols and recommend combining resistance and aerobic training with a proper diet [11]. In our research, we have shown that the lack of an adequate level of physical activity increases body weight and increases BMI by an average of 0.55 points.

Our study showed that in the BE group, the WHR index decreased, while it increased in the PED group, but this change was not statistically significant. As for the thickness of the triceps skin fold, the results were lower for the exercise groups and slightly higher for the CO group. Ruiz-Montero et al. showed that after 24 weeks of Pilates exercises and aerobic exercise, there was a statistically significant improvement in anthropometric parameters. However, the authors showed that in the case of skin fold, some participants had higher scores, while others showed a decrease in their scores. In addition, the authors showed that the waist circumference of the participants did not decrease and the WHR index was higher after the examination [1]. In their study, Kirton et al. did not observe significant differences after 12 weeks of physical exercise intervention in the WHR index between people in a group with individualized exercises and people exercising in a group. The authors suggest that individual exercise in older people with a low activity index is more beneficial in improving cardio-respiratory fitness than in reducing body weight [63]. Rugbeer et al. assessed the effect of group exercise among older patients on the anthropometric profile, demonstrating a decrease in the thickness of the skin fold in the group that exercised three times a week. The authors suggest that exercising three times a week could therefore protect against excessive body fat, which reduces the risk of cardiovascular disease in older patients in long-term care facilities [64].

Our research showed a significant increase in the AMA index in the PED group compared to the BE group and a decrease in the CO group. The decrease in the value of the variables evaluating muscle mass in older age groups is alarming because the loss of muscle mass affects the limitation of functional fitness in everyday life. Muscle strength and muscle mass are prognostic measurements of independence and mobility in older patients [65]. Additionally, Sigh et al. showed a relationship between anthropometric parameters, including the AMA index, and depressive symptoms among older patients. The authors also showed that AMA is a better indicator of health monitoring compared to the traditionally used BMI and WHR indicators [66].

Despite the extensive literature on the impact of physical activity on older patients’ physical fitness and anthropometric measurements, there are few reports focusing on functionally limited older patients. Older people, especially those with physical limitations living in nursing homes, need more support and motivation to take up physical exercise. Physical activity of older patients should be comprehensive, including moderate-intensity aerobic exercise, strengthening exercises, and exercises that improve balance. Both physical exercises with elements of dancing and basic exercise are in line with the above recommendations and are a valuable form of exercise for functionally limited older patients. In addition, exercising with music increases motivation and improves well-being [67].

One of the biggest problems related to the aging of society and low awareness of the need for physical activity in older patients is the progressive limitation of fitness in everyday activities, which in turn increases the degree of dependence on third parties. Our own research shows that older patients taking up physical exercise contributes to the extension of the period of independence in everyday activities, which is reflected in an improvement of the quality and satisfaction of life in older patients. There is a need to look for effective and inexpensive solutions to improve the functional fitness and quality of life of older patients. The results of these studies confirm the need for systematic rehabilitation in order to avoid functional and psychosocial disability. The practical aspect of the above research is the identification of simple and inexpensive exercise programs for older patients with reduced mobility.

This study had some limitations. Firstly, the groups were not initially homogeneous in terms of all parameters tested. Secondly, the authors did not carry out an assessment after 36 weeks. When designing further studies, sampling points will be scheduled at 6- and 12-month marks in order to assess long-term effects.

## 5. Conclusions

In conclusion, a 12-week program of group physical exercises, both physical exercises with elements of dancing and basic exercise, improve physical fitness indicators and anthropometric indicators.

Functionally limited older patients living in nursing homes could participate in group exercise programs tailored to their individual needs and abilities. In addition, building on this multi-component strength, balance, and endurance program, further research will help formulate important recommendations for action and guidance for promoting the health of nursing home residents.

In addition, the results of our study contributed to the introduction of systematic dance therapy using the described intervention in nursing homes in the Podkarpacie region (south-eastern Poland). This is the first step in developing proven intervention procedures.

## Figures and Tables

**Table 1:** Sociodemographic and clinical characteristics of the participants.

Variable		Number (%) Mean (SD)				
		All	Group BE (n = 56)	Group PED (n = 57)	Group CO (n = 56)	*p*-Value
Sociodemographic and clinical						
Sex	Female	98 (57.99)	28 (50.00)	37 (64.91)	33 (58.93)	*p* = 0.271 ^c^
	Male	71 (42.01)	28 (50.00)	20 (35.09)	23 (41.07)	
Marital status	Married	18 (10.65)	9 (16.07)	2 (3.51)	7 (12.50)	*p* = 0.254 ^c^
	Widow/widower	74 (43.79)	26 (46.43)	28 (49.12)	20 (35.71)	
	Divorced	28 (16.57)	9 (16.07)	10 (17.54)	9 (16.07)	
	Single	49 (28.99)	12 (21.43)	17 (29.82)	20 (35.71)	
Education	Basic	52 (30.77)	19 (33.93)	9 (15.79)	24 (42.86)	*p* = 0.028 ^c^ CO < BE, PED
	Vocational	100(59.71)	33 (58.93)	41 (71.93)	26 (46.43)	
	Higher	17 (10.06)	4 (7.14)	7 (12.28)	6 (10.71)	
Length of NH residency	>12 moths	4 (2.37)	1 (1.79)	2 (3.51)	1 (1.79)	*p* = 0.286 ^c^
	1–5 years	76 (44.97)	27 (48.21)	27 (47.37)	22 (39.29)	
	6–10 years	75 (44.38)	26 (46.43)	25 (43.86)	24 (42.86)	
	over 10 years	14 (8.28)	2 (3.57)	3 (5.26)	9 (16.07)	
Chronic disease	Cardiovascular	89 (52.66)	29 (51.79)	29 (50.88)	31 (55.36)	*p* = 0.881 ^c^
	Pulmonary	118 (69.82)	39 (69.64)	41 (71.93)	38 (67.86)	*p* = 0.894 ^c^
	Neurological	33 (19.53)	15 (26.79)	8 (14.04)	10 (17.86)	*p* = 0.215 ^c^
	Musculoskeletal	27 (15.98)	8 (14.29)	11 (19.30)	8 (14.29)	*p* = 0.702 ^c^
	Urinary system	115 (68.05)	40 (71.43)	39 (68.42)	36 (64.29)	*p* = 0.718 ^c^
	Otological	58 (34.32)	20 (35.71)	16 (28.07)	22 (39.29)	*p* = 0.439 ^c^
	Ophthalmological	85 (50.30)	21 (37.50)	32 (56.14)	32 (57.14)	*p* = 0.064 ^c^
	Digestive system	42 (24.85)	16 (28.57)	10 (17.54)	16 (28.57)	*p* = 0.292 ^c^
Number of meals consumed per day		3.11 (0.79)	3.13 (0.76)	3.11 (0.84)	3.11 (0.78)	*p* = 0.997 ^b^
Number of glasses of water drunk per day		3.04(1.14)	3.00 (1.21)	3.23 (1.09)	2.87 (1.13)	*p* = 0.279 ^b^
Subjective assessment of the nutritional status	Being malnourished	77 (45.56)	23 (41.07)	30 (52.63)	24 (42.86)	
	Can notasses	38 (22.49)	11 (19.64)	9 (15.79)	18 (32.14)	*p* = 0.170 ^c^
	Properly nourished	54 (31.95)	22 (39.29)	18 (31.58)	14 (25.00)	
Groups of food products	At least one milk product per day	132 (78.11)	37 (66.07)	49 (85.96)	46 (82.14)	*p* = 0.026 ^c^ BE < PED, CO
	Legumes or eggs at least two times a week	105 (62.13)	33 (58.93)	32 (56.14)	40 (71.43)	*p* = 0.205 ^c^
	Meat at least once a day	134 (79.29)	46 (82.14)	48 (84.21)	40 (71.43)	*p* = 0.199 ^c^
	Fruit and vegetables—Two or more meals a day	133 (78.70)	45 (80.36)	44 (77.19)	44 (78.57)	*p* = 0.919 ^c^
Age [years]		74.40 (7.45)	74.40 (7.71)	73.80 (7.35)	75.00 (7.38)	*p* = 0.717 ^b^
Body mass [kg]		71.62 (14.99)	74.85 (15.02)	73.45 (14.16)	66.44 (14.71)	0.0085 ^b^ CO < BE, PED
Height [cm]		70.92 (14.94)	161.80 (10.91)	161.69 (9.58)	161.38 (10.03)	*p* = 0.975 ^b^
Dominant limb	Right	141 (83.40)	48 (85.71)	51 (89.47)	42 (75.00)	*p* = 0.178 ^c^
	Left	28 (16.60)	8 (14.29)	6 (10.53)	14 (25.00)	
**Outcome**						
Muscle strength	HGS R [kg]	12.81 (5.27)	12.71 (5.34)	12.61 (5.15)	13.01 (5.41)	*p* = 0.830 ^a^
	HGS L [kg]	11.20 (4.41)	11.10 (4.27)	11.00 (4.08)	11.60 (4.89)	*p* = 0.676 ^b^
Manual endurance of the upper limb	ACT [x]	9.17 (3.68)	9.11 (3.77)	9.11 (9.95)	9.29 (3.33)	*p* = 0.823 ^b^
Functional Assessment	IB	54.03 (11.69)	54.01 (12.62)	54.90 (12.19)	53.80 (10.31)	*p* = 0.798 ^b^
Body Balance Assessment	BBS	12.41 (5.61)	12.26 (6.38)	12.90 (5.17)	12.20 (5.29)	*p* = 0.962 ^b^
Triceps skinfold [mm]		20.78 (8.89)	21.18 (9.23)	21.44 (9.19)	19.71 (8.26)	*p* = 0.795 ^b^
BMI [kg/m^2^]		27.49 (4.45)	28.30 (4.45)	27.84 (3.93)	26.31 (4.76)	*p* = 0.045 ^a^ CO < BE
	Underweight	35 (20.71)	8 (14.29)	9 (15.79)	18 (32.14)	
	Normal weight	80 (47.34)	24 (42.86)	30 (52.63)	26 (46.43)	
	Overweight	54 (31.95)	24 (42.86)	18 (31.58)	12 (21.43)	
AMA		3846.98 (1529.07)	4048.67 (1507.51)	3322.94 (1438.45)	4178.68 (1524.57)	*p* = 0.003 ^b^ PED < BE, CO
	good general conditions	127 (75.15)	42 (75.00)	43 (75.44)	42 (75.00)	
	malnutrition	42 (24.85)	14 (25.00)	14 (24.56)	14 (25.00)	
WHR		0.999 (0.07)	1.023 (0.07)	0.977 (0.05)	0.997 (0.08)	*p* = 0.002 ^b^ PED < BE
	abdominal obesity	157 (92.90)	56 (100)	52 (91.23)	49 (87.50)	
	gluteofemoral obesity	12 (7.10)	-	5 (8.77)	7 (12.50)	

BE, basic exercises group; PED, physical exercises with elements of dancing group; CO, control group; ^a^, one-way ANOVA; ^b^, Kruskal–Wallis test; ^c^, Chi-square test of independence; SD, standard deviation; BMI, body mass index; HGS_R_, right-hand grip strength; HGS_L_, left-hand grip strength; ACT, arm curl test; BI, Barthel Index; BBS, Berg Balance Scale; AMA, arm muscle area; WHR, waist-to-hip ratio.

**Table 2:** Results of the effects in the study groups between the baseline and the 12-week timepoint.

Variable		Differences between Time at Baseline and after 12 Weeks of the Intervention Mean (SD)								
		Group BE (n = 52)			Group PED (n = 54)			Group CO (n = 50)		
		Baseline	after 12 Weeks	*p*	Baseline	after 12 Weeks	*p*	Baseline	after 12 Weeks	*p*
**Muscle strength**	**HGS R [kg]**	12.5 (5.23)	18.1 (5.69)	<0.0001 ^d^	12.6 (5.28)	19.1 (4.51)	<0.0001 ^e^	12.7 (5.34)	9.2 (3.70)	<0.0001 ^e^
	**HGS L [kg]**	10.8 (3.93)	17.4 (4.59)	<0.0001 ^d^	10.8 (4.08)	18.5 (4.12)	<0.0001 ^d^	11.3 (4.97)	10.9 (5.01)	<0.0001 ^e^
**Manual endurance of the upper limb**	**ACT [x]**	9.0 (3.81)	11.6 (3.83)	<0.0001 ^e^	9.0 (4.01)	12.7 (3.96)	<0.0001 ^e^	9.6 (3.24)	9.1 (3.18)	0.0028 ^e^
**Functional Assessment**	**IB**	54.5 (12.50)	55.1 (11.86)	0.1925 ^e^	54.3 (12.15)	58.2 (9.91)	<0.0001 ^e^	54.3 (12.15)	53.3 (9.46)	0.0401 ^e^
**Body Balance Assessment**	**BBS**	12.5 (6.27)	14.4 (5.71)	<0.0001 ^e^	12.7 (5.18)	14.4 (5.71)	<0.0001 ^e^	11.8 (5.37)	11.4 (5.64)	0.0076 ^e^
**Body mass [kg]**		74.0 (14.33)	71.5 (14.00)	<0.0001 ^e^	72.9 (14.01)	69.4 (13.30)	<0.0001 ^e^	67.2 (15.03)	68.6 (15.18)	<0.0001 ^e^
**BMI [kg/m^2^]**		28.2 (4.22)	27.3 (4.06)	<0.0001 ^e^	27.8 (4.03)	26.3 (4.05)	<0.0001 ^e^	26.6 (4.83)	27.2 (4.86)	<0.0001 ^e^
**Triceps skinfold**	**TSF**	21.3 (9.45)	19.3 (8.61)	<0.0001 ^e^	21.6 (9.23)	18.6 (8.31)	<0.0001 ^e^	19.4 (8.43)	20.3 (8.76)	0.0074 ^e^
**WHR**		1.022 (0.07)	0.994 (0.14)	0.0032 ^e^	0.975 (0.05)	0.994 (0.12)	0.6389 ^e^	0.998 (0.08)	1.001 (0.08)	0.0080 ^e^
**AMA**		4036.7 (1542.03)	4477.9 (1669.95)	<0.0001 ^e^	3327.2 (1464.60)	4212.6 (1793.25)	<0.0001 ^e^	4298.4 (1543.69)	4151.7 (1468.08)	0.0002 ^e^

BE, basic exercises group; PED, physical exercises with elements of dancing group; CO, control group; BMI, body mass index; HGS_R_, right-hand grip strength; HGS_L_, left-hand grip strength; ACT, arm curl test; BI, Barthel Index; BBS, Berg Balance Scale; AMA, muscular area of the arm; WHR, waist-to-hip ratio; ^d^, Student’s *t*-test (dependent samples); ^e^, Wilcoxon test; SD, standard deviation.

**Table 3:** Mean difference scores for each group across time.

Variable		Mean Change from Baseline (95% CI)		
		Baseline—12 Weeks		
		Group BE (n = 52)	Group PED (n = 54)	Group CO (n = 50)
Muscle strength	HGS R [kg]	5.54 (4.89; 6.19)	6.54 (5.81; 7.28)	−0.77 (−1.06; −0.48)
	HGS L [kg]	6.60 (6.08; 7.11)	7.74 (7.11; 8.36)	−0.40 (−0.56; −0.24)
Manual endurance of the upper limb	ACT [x]	2.56 (1.98; 3.13)	3.63 (3.08; 4.18)	−0.52 (−0.86; −0.18)
Functional Assessment	IB	0.58 (−0.23; 1.39)	3.98 (2.67; 5.29)	−1.40 (−2.59; −0.21)
Body Balance Assessment	BBS	1.90 (1.51; 2.30)	2.67 (2.29; 3.05)	−0.40 (−0.66; −0.14)
Body mass [kg]		−2.50 (−2.88; −2.11)	−3.50 (−3.87; −3.12)	1.40 (0.95; 1.86)
BMI [kg/m^2^]		−0.96 (−1.12; −0.81)	−1.51 (−1.89; −1.13)	0.55 (0.38; 0.73)
Triceps skinfold	TSF	−2.00 (−2.49; −1.51)	−3.00 (−3.64; −2.36)	0.94 (0.25; 1.63)
WHR		−0.03 (−0.06; 0.01)	0.02 (−0.01; 0.05)	0.003 (0.001; 0.005)
AMA		441.13 (349.81; 532.46)	885.32 (707.42; 1063.23)	−146.66 (−433.98; 140.67)

BE, basic exercises group; PED, physical exercises with elements of dancing group; CO, control group; BMI, body mass index; HGS_R_, right-hand grip strength; HGS_L_, left-hand grip strength; ACT, arm curl test; BI, Barthel Index; BBS, Berg Balance Scale; AMA, arm muscle area; WHR, waist-to-hip ratio.

**Table 4:** Between-group comparisons of changes after 12 weeks.

Variable		After 12 Weeks		
		Group PED vs. BE	Group PED vs. CO	Group BE vs. CO
Muscle strength	HGS R [kg]	*p* = 0.002 ^g^	*p* <0.001 ^g^	*p* < 0.001 ^g^
	HGS L [kg]	*p* = 0.006 ^f^	*p* <0.001 ^g^	*p* < 0.001 ^g^
Manual endurance of the upper limb	ACT [x]	*p* = 0.024 ^g^	*p* < 0.001 ^g^	*p* < 0.001 ^g^
Functional Assessment	IB	*p* = 0.001 ^g^	*p* < 0.001 ^g^	*p* < 0.001 ^g^
Body Balance Assessment	BBS	*p* = 0.008 ^g^	*p* < 0.001 ^g^	*p* < 0.001 ^g^
Body mass [kg]		*p* = 0.001 ^g^	*p* < 0.001 ^g^	*p* < 0.001 ^g^
BMI [kg/m^2^]		*p* = 0.001 ^g^	*p* < 0.001 ^g^	*P* < 0.001 ^g^
Triceps skinfold [mm]	TSF	*p* = 0.018 ^g^	*p* < 0.001 ^g^	*p* < 0.001 ^g^
WHR		*p* = 0.008 ^g^	*p* = 0.132 ^g^	*p* = 0.001 ^g^
AMA		*p* < 0.001 ^g^	*p* < 0.001 ^g^	*p* < 0.001 ^g^

BE, basic exercises group; PED, physical exercises with elements of dancing group; CO, control group; BMI, body mass index; HGS_R_, right-hand grip strength; HGS_L_, left-hand grip strength; ACT, arm curl test; BI, Barthel Index; BBS, Berg Balance Scale; AMA, arm muscle area; WHR, waist-to-hip ratio; ^f^, Student’s *t*-test (independent samples); ^g^ Mann–Whitney U Test.

## Data Availability

The datasets used and analyzed in the current study are available from the corresponding author upon reasonable request.
